# Approach to Malnutrition and Oral Nutrition Therapy in Adults with IBD: What to Consider

**DOI:** 10.3390/nu18020204

**Published:** 2026-01-08

**Authors:** Jessica Sosio, Mark Zemanek, Lindsey Anne Russell

**Affiliations:** 1 Department of Gastroenterology, Hepatology, and Nutrition, Cleveland Clinic, Cleveland, OH 44195, USA; 2 Cleveland Clinic Lerner College of Medicine, Case Western Reserve University, Cleveland, OH 44106, USA

**Keywords:** inflammatory bowel disease, malnutrition, malnutrition assessment, oral nutritional supplementation, vitamin supplementation

## Abstract

Inflammatory Bowel disease (IBD), including Crohn’s disease (CD) and ulcerative colitis (UC), are chronic gastrointestinal conditions that are prone to malnutrition due to poor oral intake, intestinal compromise of nutrient absorption, and increase in metabolic demand. Screening and diagnosing malnutrition in this population is necessary to treat and prevent worsening malnutrition. The use of Oral Nutritional Therapy (ONS) can provide the macronutrients that patients need to maintain their nutrition, however their role in within stages of diseases, active disease, remission, perioperative, and maintenance in relation to other nutritional therapies, such as enteral nutrition or parenteral nutrition, is unclear. This review will highlight the principles of diagnosing malnutrition, the evidence of ONS in disease and remission states, and the role of oral vitamins in the management of IBD.

## 1. Introduction

IBD is a chronic inflammatory condition affecting the gastrointestinal tract, with North America having the highest age-standardized incidence rates of IBD with climbing incidence rates in developing countries, partly due to the westernization of diets [1,2]. Patients with IBD often experience complications in the setting of malnutrition that can lead to multiple co-morbidities leading to high healthcare costs, averaging $9000–12,000 per patient annually [3,4]. Malnutrition is highly prevalent in patients with IBD and can affect up to 85% of patients with CD and up to 60% of patients with UC [5]. While diet has been explored extensively to improve overall health status in patients with IBD, those with malnutrition may benefit from oral nutritional supplements (ONS), as well as vitamin and mineral supplementation. ONS has been shown to be valuable for patients to meet their nutritional needs prior to immediately resorting to more invasive therapies such as enteral nutrition (EN) or parenteral nutrition (PN). ONS can provide an additional 600 kcal/day, which can be especially significant for patients with IBD in remission when energy-protein intake is insufficient to meet requirements [6]. Vitamins and supplements also play an important role as they can further optimize nutritional status perioperatively, treat anemia, or prevent osteoporosis. It is estimated that the cumulative rate of surgery for patients with CD within 30 years of symptom onset can be as high as 94.5% [7]. Therefore, it is essential to understand how best to optimize modifiable factors such as malnutrition, anemia, frailty, and medications during the perioperative period to help prevent post-surgical complications [7]. This review highlights some of the more recent evidence for evaluating malnutrition, the use of ONS, vitamin supplementation, and other supplements available to date in managing various stages of IBD.

A comprehensive literature search was conducted in PubMed, Embase, and Medline to identify relevant studies on malnutrition assessment, ONS, and vitamins in IBD. Studies were also identified though manual searches within references from studies, review articles, and clinical guidelines. The search terms included inflammatory bowel disease, Crohn’s disease, ulcerative colitis, malnutrition screening, malnutrition assessment, body composition, obesity, and oral nutritional supplementation, ONS, oral nutrition support, perioperative, supplementation, nutrition, vitamins, (see Supplementary Materials). Peer-reviewed studies and manuscripts assessing ONS, malnutrition assessment, and obesity in adult patients with IBD were included at the discretion of the authors.

## 2. Malnutrition Assessment

Malnutrition affects up to 85% of patients with IBD and may lead to adverse consequences including a higher rate of complications, a reduced response to therapeutic agents, prolonged hospital stays, mortality, and an overall decrease in quality of life [8,9]. Per the European Society for Clinical Nutrition and Metabolism (ESPEN), IBD patients are especially at risk due to inadequate energy intake, utilization of elimination diets, and alterations in bowel function leading to diarrhea [9]. Due to this high prevalence of malnutrition, all IBD patients should be screened and assessed accordingly [8]. Specifically, per ESPEN guidelines, all IBD patients should be screened for malnutrition at the time of diagnosis and thereafter on a regular basis. If active disease is present, an assessment or re-assessment for malnutrition is warranted [10]. The Academy of Nutrition and Dietetics and the American Society for Parenteral and Enteral Nutrition (ASPEN) describe malnutrition screening as a tool to be used to identify patients at high risk of malnutrition with a need to undergo further assessment and potential nutritional intervention [11]. Malnutrition assessment is defined as a comprehensive evaluation that looks at different surrogate measures to interpret nutritional status and utilizes body composition and physiologic function to support a potential diagnosis of malnutrition [11].

There are numerous screening tools to detect malnutrition: the Malnutrition Screening Test (MST), the Malnutrition Universal Screening Tool (MUST), the Mini Nutritional Assessment (MNA) tool, the Nutrition Screening 2002 (NRS2002) tool, and the Nutritional Risk Index (NRI), as well as IBD-specific tools such as the Saskatchewan IBD-Nutrition Risk (SASKIBD-NR) tool and the Malnutrition Inflammation Risk Tool (MIRT) [9,12] (Table 1). Specifically, MIRT, MNA, MUST, NRI, and NRS2002 include similar parameters, such as body composition or body mass index (BMI), and disease activity in terms of clinical symptomatology or biochemical markers (albumin for NRI and C-reactive protein for MIRT). Except for NRI, this subgroup also included unintentional weight loss over a period of 3 months. MNA, MUST, and NRS2002 further included changes in food intake. Additionally, depression and the presence of mobility limitations were associated with a higher risk of malnutrition per MNA [9].

**Table 1:** Malnutrition Screening in IBD patients [9,12].

Malnutrition Screening Tool	Type	Key Parameters Assessed	IBD-Specific Considerations/Notes
Malnutrition Screening Test (MST)	General screening	Unintentional weight loss, decreased appetite	High predictive accuracy vs. GLIM in IBD [9]
Malnutrition Universal Screening Tool (MUST)	General screening	BMI, unintentional weight loss, acute disease effect	Strong accuracy compared with GLIM in IBD [9]
Mini Nutritional Assessment (MNA)	General screening	BMI, weight loss, intake, mobility, depression	Includes psychosocial factors; lower accuracy in IBD [9]
Nutrition Risk Screening 2002 (NRS-2002)	General screening	BMI, weight loss, reduced intake, disease severity	Moderate performance in IBD [9,12]
Nutritional Risk Index (NRI)	General screening	Serum albumin, weight change	Affected by inflammation; no intake assessment [9]
Malnutrition Inflammation Risk Tool (MIRT)	General screening	BMI, CRP, disease activity	Moderate accuracy vs. GLIM [9,12]
Saskatchewan IBD-Nutrition Risk (SASKIBD-NR)	Screening (IBD-specific)	BMI, weight loss, intake, GI symptoms	Lower accuracy vs. GLIM [9,12]
Nutrition Screening-IBD (NS-IBD)	Screening (perioperative, IBD-specific)	BMI, weight loss, prior surgery, diarrhea/ileostomy, GI symptoms	High sensitivity; lower specificity vs. GLIM [12]

IBD: inflammatory bowel disease; BMI: body mass index; CRP: C-reactive protein; and GLIM: Global Leadership Initiative on Malnutrition. Table summary aided by ChatGPT.

At this time, there is no gold standard to diagnose malnutrition. Diagnostic tools include the Subjective Global Assessment (SGA) tool [8], the criteria from the World Health Organization (WHO)-defined BMI cut-off value, and the ESPEN criteria of malnutrition in 2015, which have been available for a few years (Table 2). More recently, the Global Leadership Initiative on Malnutrition (GLIM) criteria were established with input from major nutritional societies including ESPEN, ASPEN, the Parenteral and Enteral Nutrition of Asia (PENSA), and the Federation Latinoamericana de Terapia Nutricional (FELANPE) in effort to reach a consensus in the standardization of the criteria for a diagnosis of malnutrition [8,13]. The GLIM assessment is made up of two mandatory criteria, phenotypic and etiological. Phenotypic criteria determine the severity of malnutrition in terms of body composition and weight, while etiological criteria highlight the underlying cause leading to malnutrition, such as inflammation [9,13]. In terms of screening applicability in the IBD population, a retrospective study in 2024 comparing different screening tools against GLIM criteria found that MST and MUST had the highest level of accuracy in predicting malnutrition [9]. SASKIBD-NR and MNA did not perform as accurately [9]. Perioperatively, a prospective study in 2021 compared a novel screening tool named NS-IBD to SASKIBD-NR, MUST, MST, NRS-2002, and MIRT; all compared in performance against the GLIM criteria [12]. NS-IBD was found to perform well in terms of sensitivity and accuracy, but not as well in terms of specificity. Of note, the parameters included in NS-IBD were BMI, UWL, previous abdominal IBD surgery, presence of chronic diarrhea or ileostomy, and presence of certain gastrointestinal symptoms [12]. In terms of diagnosis of malnutrition, a prospective study in 2022 analyzing the concordance of WHO, SGA, ESPEN, and GLIM criteria in IBD patients found that the GLIM criteria showed good concordance with ESPEN and fair concordance with SGA; this suggests that the GLIM criteria is a more reliable diagnostic tool [8]. Perioperatively, the GLIM criteria also appeared to detect malnutrition well, but room for the development of a higher-performing tool is likely needed [13].

**Table 2:** Malnutrition Assessment [8,9,13].

Tool/Criteria	Core Components	Strengths/Limitations in IBD
Subjective Global Assessment (SGA)	Weight change, intake, GI symptoms, physical exam	Subjective; fair concordance with GLIM [8]
WHO BMI Criteria	BMI cut-off values	Simple; limited detection of inflammatory malnutrition [8]
ESPEN Criteria (2015)	BMI, weight loss, fat-free mass index	Good concordance with GLIM [8,13]
GLIM Criteria	Phenotypic + etiologic criteria	Reliable in IBD; requires prior screening [8,9,13]

WHO: World Health Organization; BMI: body mass index; ESPEN: European Society for Clinical Nutrition and Metabolism; and GLIM: Global Leadership Initiative on Malnutrition. Table summary aided by ChatGPT 2025.

Obesity in patients with IBD has become more prevalent over the years; a retrospective US study on 581 IBD patients published in 2015 identified obesity in 32.7% of its population [14]. This creates challenges in the identification of malnutrition due to hidden deficits in lean muscle mass [10]. The severity of obesity adds an extra layer of complexity, with class II (BMI 35 to less than 40) and III (BMI 40 or greater) facing more challenges in assessing lean muscle mass accurately. Several tools are available to assess body composition and evaluate muscle mass, including electrical bioimpedance analysis (BIA), dual-energy X-ray absorptiometry (DXA), magnetic resonance imaging (MRI), computed tomography (CT), and ultrasonography (US). BIA and DEXA have less radiation exposure, but may not always be feasible to perform in patients with a BMI  ≥  40 kg/m^2^. Both MRI and CT can be used to evaluate skeletal muscle, but they have limitations. MRI may not be feasible in patients with large body size, and CT utilizes high radiation levels which can pose a risk to health; however, existing CT for IBD patients can be used to assess body composition over time points. US is a valuable tool, but it can be technically challenging to perform [15]. Ultimately, muscle mass estimation in obese patients is dependent on equipment availability and clinician comfort in interpreting imaging findings, and has been up and coming for use to complete a thorough assessment on body composition in the context of malnutrition.

## 3. Oral Nutritional Supplements in IBD

Dietary modifications involving adherence to diets such as plant-based, Mediterranean, and low-FODMAP diets have been instrumental in managing the symptoms of IBD [11]. The use of ONS as a therapeutic strategy has a critical role in helping patients meet their nutritional needs by supplying highly concentrated, easy-to-digest, and bioavailable macro- and micronutrients [5]. They are part of a broader category of foods labeled as foods for special medical purposes (FSMPs), which are formulated for the dietary management of patients suffering from disorders that impact adequate nutrition. However, unlike FSMPs, they are defined as foods specially formulated for patients with malnutrition and are intended to be used only under medical supervision [6]. Their composition varies by country due to different regulations. Additionally, due to a lack of legislature defining the exact compositions of ONS, manufacturers are tasked with establishing the target audience and indication for the use of each ONS.

**Figure 1:**
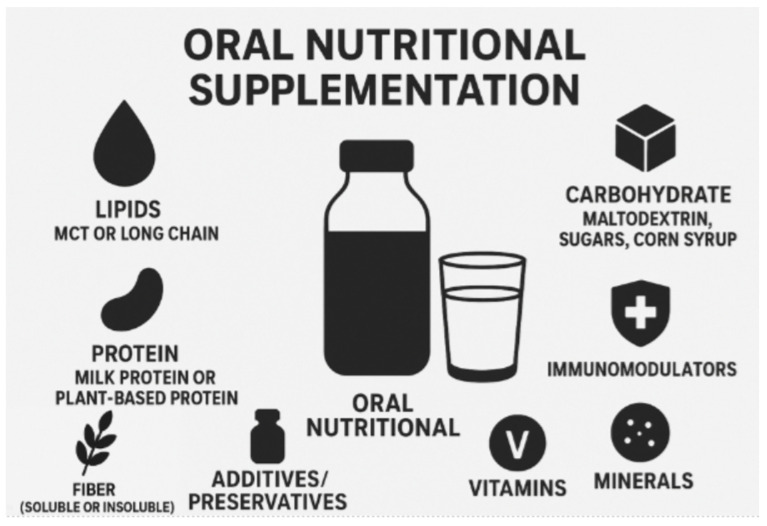
Components in Oral Nutritional Supplements to consider for recommendation. AI image generator ChatGPT 2025 was used.

A wide variety of products exist on the market, offering multiple options in terms of consistency, volume, flavor, and nutritional composition (Figure 1). The spectrum for consistency ranges from powder to liquid with various viscosity formulations. Specialized nutritional products containing growth factors or reduced emulsifiers have not shown significant positive effects overall and, surprisingly, potentially problematic additives such as carrageenan or modified starch did not appear to negatively affect CD patients, shown in a recent review on various ONS available [5]. Furthermore, ONS containing additives such as immunomodulators like omega 3 fatty acids, muscle mass preservation such as β-hydroxy-β-methylbutyrate (β-HMB), and arginine and glutamine, have not have robust evidence for their routine use as per guidelines [5]. A systematic review in 2020 found that glutamine supplementation had no effect on disease course or activity, anthropometrics, intestinal symptoms, biochemical parameters, and inflammation markers in three studies that assessed oral administration of glutamine, however the sample size was small (28 patients total in treated group) [16]. In practice, medium-chain triglycerides (MCTs) and amino acid-based supplements have been used to support fat absorption to minimize gastrointestinal stress [5]. A prospective study in 2019 analyzed the impact of ONS containing transforming growth factor-beta 2 (TGF-β2), a protein compound that can act to modulate inflammation and help mucosal repair, on gut inflammation, and found histologic improvement of disease as well as reduced inflammation based on CRPs with their use [17].

There are very few IBD-specific ONS, but no standard formulation requirements are in place to offer a therapy that is specifically tailored to IBD on a global scale. However, despite the variety of non-macronutrient components, all ONS share high-calorie and high-protein properties to help support the nutritional needs of IBD patients across different disease stages. ESPEN recommends strategic use based on disease activity and certain clinical circumstances including preoperatively, during recovery, and long-term for those unable to meet energy requirements (Table 3) [5].

**Table 3:** Oral Nutritional Supplements (ONS) Across Stages of Inflammatory Bowel Disease (IBD) [5,6,18,19,20].

Disease Stage	ONS Characteristics	Clinical Rationale and Evidence
Active Disease	High-protein (1.2–1.5 g/kg/day) and high-calorie formulations; MCT-containing, hypolipidic or lipid-free for easier fat absorption; lactose-free, fiber-free, low-residue to reduce GI irritation; may include TGF-β2–enriched products for mucosal repair	Addresses elevated protein needs from inflammation, malabsorption, and catabolism. TGF-β2 ONS improve mucosal histology and reduce CRPs [18]. Fiber- and lactose-free products minimize GI distress [6]. ESPEN recommends ONS during active disease and hospitalization [5].
Early Remission	High-protein, high-calorie, lactose-free, fiber-free ONS; semi-elemental or polymeric formulations as tolerated	Supports recovery from acute inflammation while maintaining nutritional adequacy. Aids healing during lingering mucosal inflammation [5,6].
Late Remission	High-protein, high-calorie, low-fiber, lactose-reduced ONS; may include fermentable prebiotic fibers (inulin, FOS, GOS)	Promotes intestinal recovery and restoration of healthy microbiota. Gradual fiber reintroduction enhances tolerance and microbial diversity [5].
Maintenance/ Quiescent Disease	Limited ONS role; used when oral intake or weight maintenance is inadequate. Partial enteral nutrition (PEN) may complement solid diets	Long-term management emphasizes diet (e.g., Mediterranean diet) over ONS. EEN/PEN beneficial primarily in CD, not UC [19]. ONS may support patients during stress or appetite loss.
Peri-operative Period	Pre-operative: High-protein ONS (≥18 g/dose), 2–3× daily for ≥7 days. Post-operative: Continue high-protein ONS ≥ 7 days, especially in malnourished or sarcopenic patients. May use immunonutrient-enriched formulas (arginine, ω-3 FA, glutamine, RNA)	Pre-operative ONS reduce postoperative complications and enhance recovery [6]. Immunonutrient ONS shown to improve oxidative stress and gut barrier recovery in CD [21]. ESPEN suggests delaying elective surgery 7–14 days for preoperative optimization [8]. If ONS insufficient, use exclusive enteral nutrition (EEN) to improve surgical outcomes [22]. In severe malnutrition, consider diverting procedures to allow continued nutritional therapy [8].

MCT: medium-chain triglycerides; TGF-β2: transforming growth factor-beta 2; ONS: oral nutrition supplements; FOS: fructooligosaccharides; GOS: galactooligosaccharides; ω-3 FA: ω-3 fatty acids; RNA: ribonucleic acid; CRP: C-reactive protein; ESPEN: European Society for Clinical Nutrition and Metabolism; EEN: exclusive enteral nutrition; and PEN: partial enteral nutrition. Table summary aided by ChatGPT 2025.

### 3.1. Active Disease

During the acute inflammatory phase, protein requirements are increased due to inadequate dietary intake, intestinal loss of nutrients, and medication-induced changes. Those additional protein needs are compounded by inflammatory-driven catabolism, leading to proteolysis. In this state, protein needs can reach up to 1.2–1.5 g/kg/day in adults [5,6]. A recent article in 2024 described the use of non-IBD specific MCT-containing, hypolipidic, and lipid-free ONS for the active patients [6]. Of note, almost all ONS used were fiber-free, and all products were lactose-free, likely due to accommodating the inflamed bowel [6]. Low-residue formulations may also minimize gastrointestinal symptoms during clinical flare-ups [5]. A prospective study in 2019 analyzed the impact of ONS containing TGF-β2, a protein compound that can act to modulate inflammation and help mucosal repair, on gut inflammation, and found histologic improvement of disease as well as reduced inflammation based on CRP levels in 13 patients with CD [17].

A cross-sectional study, which utilized a survey distributed to physicians and dieticians during the 17th Congress of European Crohn’s and Colitis Organization (ECCO) in 2022, revealed that ONS with easy-to-digest foods was the preferred nutritional therapy option during the first 3 days of an acute flare-up in both CD and UC [18]. Exclusive use of ONS was preferentially used in CD [18]. It is important to note that the providers recommending such strategies were mostly gastroenterologists and dieticians, and a small minority were colorectal surgeons [18]. However, preference for ORS over PN varied worldwide, as UK providers favored ONS and Italian providers preferred PN, whereas providers from other European countries had no preference [18]. The survey also inquired about factors providers use to make changes in prescribed nutritional therapies, and parameters such as energy intake, stool frequency, inflammatory biomarkers, and appetite were found to be used as common metrics. Of those, gastroenterologists placed more value on total energy intake as the deciding factor for therapy adjustments. Only a minority of dieticians utilized inflammatory markers. Nutritional status was reported to be mostly monitored by approximate methods by all providers surveyed except for Swedish professionals, who preferred a more detailed approach featuring calculations of caloric and volume intake [18]. This survey study highlights the inconsistencies in clinical practice and presents a call to action for position statements to help guide nutritional care.

### 3.2. Remission

This stage of the disease still involves some degree of inflammation, which tends to gradually subside as the gastrointestinal tract recovers from the acute phase. An article featuring Italian FSMPs and ONS found that lactose-free and fiber-free products were useful in the early stages of remission, likely due to the lingering inflammation from the acute phase [6]. In contrast, the later stages of remission showed tolerance of products containing low fiber and lactose. Regardless of remission stage, all products were high in calories and protein to meet nutritional needs in the setting of reduced oral intake [5,6]. Specifically, fermentable fiber in the form of inulin, fructooligosaccharides (FOS), and galactooligosaccharides (GOS) were proposed to positively impact the composition of the gut microbiota [5]. Different nutritional compositions also exist for ONS, ranging from elemental, semi-elemental, and polymeric formulas, but no specific formulation appears to be more effective in the induction of remission during active disease in CD patients [5]. Generally, the role of ONS at this stage is to provide a hypercaloric and high protein formula to help maintain adequate nutrition.

### 3.3. Maintenance/Quiescent Disease

While ONS play a key role in active disease and remission, their utility in quiescent disease remains unexplored. At this stage, diet has shown to have an important role in managing both induction and maintenance, especially in CD [5]. Specifically, a study compared the consumption of solid food diets, such as the Mediterranean diet, with that of exclusive enteral nutrition (EEN) or partial enteral nutrition regarding efficacy in induction or maintenance of remission in IBD [23]. While the impact of solid food diets on induction or maintenance of remission for IBD is unclear, EEN was shown to be effective at inducing remission in patients with CD. Partial enteral nutrition may also be beneficial in patients with CD. Changes in dietary habits such as reducing refined carbohydrates or red meat alone may not be adequate to avoid clinical relapse in CD. No specific diet was identified to be helpful in patients with UC in the setting of induction or maintenance of remission [23].

### 3.4. Perioperative

In patients with a functioning gastrointestinal tract devoid of obstructive processes, ONS serve a key role in nutritional optimization prior to abdominal surgery, leading to less postoperative complications. While not specific to IBD patients, general nutritional considerations for those undrgoing abdominal surgery can be applied to IBD patients perioperatively [6]. In the preoperative period, a protein goal of 1.2 g/kg of body weight is recommended. If patients are at a particular risk of malnutrition, high-protein ONS administered for at least 7 days may help reduce poor postoperative outcomes [6]. Specifically, those ONS should provide a minimum of 18 g of protein per dose and be consumed 2–3 times daily. In the immediate postoperative period, those undergoing non-emergent planned major abdominal surgery should continue to receive ONS for an additional 7 days. Only patients with risk factors such as sarcopenia and malnutrition and those who cannot maintain appropriate energy requirements should continue ONS [6].

A recent study assessed ONS in the setting of elective surgery in 61 adults with IBD and found no significant change in body composition with ERAS protocol and targeted nutritional supplementation [24]. A pilot study in 2023 analyzed the effects of ONS enriched with immunonutrients on CD patients undergoing intestinal surgery for active disease, and found this formulation led to a shorter time to resumption of a regular diet [19]. Of note, the nutrients with immune-activating properties included biologically active nutrients such as arginine, omega-3 fatty acids, glutamine, and RNA [19]. When ONS are not sufficient to optimize patients, exclusive enteral nutrition can be an effective treatment strategy to optimize the nutritional status in malnourished patients with CD in the preoperative phase and reduce postoperative complications [21]. Per ESPEN guidelines, one may consider postponing operations for 7 to 14 days to initiate exclusive enteral nutrition allowing patients to convert from a catabolic to an anabolic state [22]. For patients with severe protein-calorie malnutrition who require emergent surgeries, diverting the anastomosis or performing an end ileostomy can be considered to allow time for additional nutritional supplementation [7].

### 3.5. General Considerations

ONS remain a valuable tool in the nutritional management of IBD, allowing patients not only to gain weight, but to also experience positive outcomes such as lowering their risk of infection, improving wound healing, and decreasing the use of healthcare resources [5] (Table 1). However, in patients unable to meet nutritional needs with a combination of diet and ONS, the typical progression is to recommend enteral nutrition first, followed by parenteral nutrition as a last resort. According to the AGA Clinical Practice Updates, there has been evidence that exclusive enteral nutrition can be effective for both clinical remission and endoscopic response in patients with CD, while enteral nutrition can be beneficial for patients as a liquid diet and a steroid sparing strategy [21]. In patients with IBD requiring additional parenteral nutrition support, customized hydration management strategies can be considered to help patients wean off parenteral nutrition. Specifically, intravenous electrolyte support and oral rehydration solutions can help to support patients with adequate hydration. Medications such as glucagon-like-peptide-2 (GLP-2) agonists can be additionally considered for patients with IBD and short bowel syndrome [21].

### 3.6. Practical Aspects of Using ONS

It is important to consider practical aspects such as choosing how and when to take ONS. Understanding when ONS are useful, choosing the right supplement, and monitoring adherence closely is essential for patient success. ONS are helpful when a patient is eating but not able to achieve goal energy needs [22]. This may include patients who are underweight, malnourished, or have a poor appetite due to illness, surgery, or injury. Addressing each patient individually allows for the best selection of ONS that may focus on calories, protein, or fiber. Acknowledging factors such as taste and texture help with patient adherence. Some strategies to enhance patient success are to take ONS between meals, rather than right before, in an effort to avoid reducing appetite. This includes sipping small amounts of ONS regularly and slowly over the course of 30–60 min. Patients may tolerate ONS better if it is chilled or consumed using a straw to reduce taste fatigue. It may be considered to incorporate ONS into foods such as smoothies or porridge. Patients may have poor adherence due to issues with taste, feeling full, or experiencing abdominal discomfort. Healthcare providers should follow patients closely to address side effects such as fullness or changes in bowel movements. Patients may need to reduce the volume and increase the frequency to better tolerate ONS. If patients report diarrhea, they can consider slowing the intake or using fiber-containing products. By contrast, if patients report symptoms of constipation, they may benefit from additional fluids. Finally, patients should be aware of storage and food safety. ONS should be shaken well before use, and once opened, they should be refrigerated and used within 24 h. In summary, providers should actively work with patients to best optimize the use of ONS. This includes emphasizing the importance of food first with supplements second, and assisting with the timing of supplements.

## 4. Vitamin Supplementation in IBD

In addition to ONS, supplementation of vitamins and micronutrients also plays an important role in controlling active disease, remission, and maintenance. Studies have been conducted to look at the benefits of oral and parenteral vitamins, micronutrients, and electrolytes in patients with protein-energy malnutrition and IBD.

### 4.1. Active Disease

In a retrospective analysis of 63 patients with active Crohn’s disease, these supplemental adjuncts were confirmed to have a beneficial effect on body weight, BMI, and serum butyryl-cholinesterase, a liver marker that reflects inflammation, malnutrition, and overall liver damage [20]. Patients in the study received both multivitamins and mineral tablets. They were given modular gluten and lactose free powder and/or ONS for a supplementary intake of 250–300 kcal/day (1–1.5 kcal/mL, 20–30% proteins, 35–45% carbohydrates, 25–45% lipids, and fiber-, lactose-, and gluten-free) [20]. Iron deficiency anemia is common in IBD and requires routine screening with a prevalence as high as 90%. It usually occurs in the setting of decreased iron absorption secondary to chronic gastrointestinal tract inflammation, resection of the small bowel where absorption occurs, malnutrition, and/or blood loss. All patients with IBD, especially those with active disease, should complete routine laboratory work with iron studies; these include blood counts, serum ferritin, transferrin saturation (TSAT), and CRPs [22]. Lab monitoring should occur every 3 months for patients with active disease. For patients with active disease, ferritin should be less than 100 µg/L. Also, a TSAT less than 20% can indicate iron deficiency anemia in active disease [25]. Patients with clinically active IBD or who are intolerant to oral iron should be treated with IV iron infusions [22]. ECCO recommends supplementing iron at the start of diagnosis and considering intravenous iron as a faster and more effective strategy [26].

### 4.2. Remission

Patients with IBD should be evaluated closely for changes in malnutrition at this stage and receive appropriate laboratory monitoring to identify potential deficiencies. For example, patients with IBD may experience chronic mucosal inflammation that affects different levels such as vitamin D, vitamin B12, and iron. These micronutrient deficiencies are important to identify early and allow for replacement to optimize nutritional status. Patients who have extensive ileal disease or prior ileal surgeries including resection or ileal pouch formation can develop vitamin B12 deficiency. Additional micronutrient, vitamin, and mineral monitoring include routine zinc, copper, folic acid, and fat-soluble vitamin deficiencies [21]. Iron deficiency anemia continues to be an important consideration to routinely monitor as patients enter remission of their disease process. Patients in remission or with mild ulcerative colitis should continue to have their iron studies monitored every 6 to 12 months. Vitamin B9, also known as folate, can also be low in patients with UC due to inadequate intake or poor intestinal absorption due to gastrointestinal inflammation [25]. This may be due to treatment with medications such as sulfasalazine or methotrexate due to their involvement in decreased B9 absorption. While omega-3 fatty acids have been shown to have potential anti-inflammatory effects in chronic conditions, there has not been any evidence for benefit in patients with IBD. Per ESPEN guidelines, there is no recommendation for the supplementation of omega-3 fatty acids for patients with IBD to maintain remission [22]. Butyrate supplementation, which can be obtained via oral route, diet, and other less conventional means, has demonstrated potential in reducing inflammation and maintaining remission per a literature review in 2023 [27].

### 4.3. Maintenance/Quiescent Disease

Iron deficiency anemia workup remains important, and IBD patients with mild anemia or clinically inactive disease can receive oral iron supplementations as a first line treatment [22]. Additionally, osteoporosis is also a common extraintestinal complication in patients with IBD. While the etiology of osteoporosis is multifactorial, it is important to monitor and supplement calcium and vitamin D levels to prevent low bone mineral density [22]. Vitamin D deficiency can be secondary to inadequate intake, impaired absorption, increased absorption due to active inflammation, or increased catabolism and renal excretion. Deficiency may be considered with a serum 25 vitamin D below 30–100 ng/mL. It is recommended by ESPEN to screen all IBD patients for vitamin D deficiency and low calcium levels [22]. This is especially important in patients receiving corticosteroid therapy, which can further increase the risk of osteoporosis. It is recommended to prescribe vitamin D3 or D2 6000 IU daily or 50,000 IU weekly for adults with vitamin D deficiency. Per ESPEN guidelines, patients with active UC who are receiving treatment with sulfasalazine, or those with macrocytosis, should be monitored for folic acid deficiency. Folic acid may also be considered in patients who are treated with methotrexate [22]. To maintain an optimal nutritional approach, it is important for providers to evaluate the state of inflammatory bowel disease. This includes understanding anatomical concerns such as strictures, fistulas, or abscesses [21]. In addition, incorporating the level of malnutrition, active inflammation, and surgical options available for candidates allows for more individualized recommendations. According to the ESPEN guidelines, there have been studies conducted on the role of omega-3 fatty acids. Unfortunately, they have shown that omega-3 fatty acid is ineffective in the maintenance of remission for patients with UC and thus should not be used. Patients with CD who have undergone resection of greater than 30 cm of distal ileum may have a prevalence of 5.6 to 38% vitamin B12 deficiency. Therefore, those with ileal involvement should be screened annually for vitamin B12 deficiency. Those with a notable clinical deficiency should be supplied with 1000 ug of vitamin B12 by intramuscular injection every other day for a week, followed by monthly injections for life [22].

### 4.4. Perioperative

In addition to ONS, there are also recommendations for perioperative micronutrients that can further enhance patient nutritional status [6]. Arginine, ω-3, and nucleotides can be beneficial in enhancing immune function, decreasing postoperative infection risk, and decreasing overall hospital length of stay [6]. Arginine is especially useful in the role of wound healing [6].

### 4.5. General Considerations

Patients with IBD who have active disease should be assessed routinely as they can be at an increased risk of nutritional deficiencies. These include iron deficiency anemia, calcium and vitamin D deficiency which can lead to bone fragility, zinc deficiencies in the setting of altered bowel function, and inadequate dietary intake [20]. Nutritional deficiencies tend to dissipate as the disease state moves towards remission and quiescency, but periodic monitoring remains important for nutritional optimization (Table 4).

## 5. Future Directions

Currently, there are very few IBD-specific ONS with no standard formulation requirements. Furthermore, most ONS on the market feature a high osmolarity, which can be problematic in IBD and lead to further gastrointestinal distress. Patient response to different formulations of ONS can be highly variable, and access and affordability is a major barrier. Given these challenges, and that most evidence is from small-number trials, further studies assessing the use of ONS formulations in larger populations across different IBD disease stages is necessary. Additionally, exploring biomarkers that can predict response to specific interventions including ONS may offer additional insight, as relying on symptoms may be challenging. Also unexplored is the effect of ONS on the microbiome [5]. A review article in 2021 proposed the need for a clinical trial testing the effects of adjunctive creatine oral supplementation in aiding disease improvement and possibly assisting in the induction of remission [28]. Creatine functions as an energy precursor that assists cells and tissues, including the intestinal epithelium, and performs essential functions. Experimentally, it was found to indirectly contribute to decreased oxidative stress, and further clinical studies are needed to see if it is beneficial in the management of IBD [28]. Finally, assessing ONS in the setting of alternating body composition, especially preserving lean body mass in IBD patients with obesity, would be particularly useful in clinical practice. Artificial intelligence (AI) is emerging as a potential tool to enhance the interpretation of imaging for body composition, with a recent observational cross-sectional study in Spain analyzing ultrasound images of skeletal muscle processed using AI to determine the muscle characteristics of malnourished patients in an inflammatory state [29]. As technology continues to evolve, more clinicians may be able to use this technology to interpret different types of imaging and better assess malnutrition and body composition.

## 6. Conclusions

Malnutrition is an important consequence of IBD, and patients may experience malnutrition due to a variety of factors such as decreased oral intake, increased energy demands, and gastrointestinal losses due to inflammation, malabsorption, or short bowel syndrome [21]. Malnutrition screening and assessment tools are necessary to use to identify patients at risk and for diagnosis. Many different tools have been developed, with some showing more promise in IBD than others, but all provide some degree of utility. Overall, performing an actual evaluation for malnutrition remains more important than the tool chosen.

Optimization of nutritional status in IBD is challenging. ONS have been shown to fill an important gap when dietary modifications alone are not enough to provide adequate nutritional needs while preventing more invasive feeding modalities; however, the majority of evidence is in the general population. While further studies are necessary to better understand how best to optimize nutritional status in patients with IBD, there is evidence for the practice of incorporating ONS and vitamin supplementation in enhancing patients with active disease, in remission, during maintenance, or in the perioperative window. Additionally, given the limited available current evidence, it is reasonable to adopt a patient-centered strategy that considers specific nutritional requirements, formula tolerance, cost, and other factors [5]. Vitamin supplementation also plays a large role and is a beneficial adjunct in successful and comprehensive nutritional management of this complex patient population.

## Figures and Tables

**Table 4:** Vitamin, Supplement, and Probiotic Use Across Stages of IBD [6,7,20,22,25,26].

Disease Stage	Vitamins and Supplements (Monitoring and Dosing)	Probiotics	Clinical Rationale and Evidence
Active Disease	Multivitamin–mineral support improves weight and nutritional markers [20]. Iron: check every 3 months (CBC, ferritin, TSAT, CRP). Ferritin < 100 µg/L or TSAT < 20% = deficiency. Prefer IV iron in active disease or oral intolerance [7,25].	Not recommended; insufficient evidence [25].	Corrects inflammation-related micronutrient loss. IV iron restores stores rapidly without GI irritation.
Remission (Early–Late)	Vitamin D: replete if <30 ng/mL (D3 6000 IU/day or 50,000 IU/week) [25]. B12: monitor in ileal disease/resection; folate for UC or sulfasalazine/methotrexate. Iron: monitor every 6–12 months. Assess zinc, copper, fat-soluble vitamins [22]. Butyrate may aid remission [26].	Possible adjunct for barrier support, but limited evidence [25].	Addresses residual deficiencies; B12, vitamin D, folate, and iron repletion support mucosal recovery and reduce relapse risk.
Maintenance/ Quiescent Disease	Oral iron for mild anemia [7]. Continue vitamin D + calcium for bone health. B12: annual screen; IM 1000 µg every other day × 1 week → monthly lifelong [7]. Folate if on sulfasalazine/methotrexate [25].	Avoid in active UC; may use selectively in remission [27].	Maintains nutritional balance and prevents osteoporosis or anemia.
Peri-operative Period	Immunonutrients (arginine, ω-3 FA, nucleotides) enhance immune response and wound healing [6]. Correct iron, D, B12, calcium ≥ 7 days pre-op.	Not recommended perioperatively (infection risk).	Arginine and ω-3 reduce infection risk and shorten recovery time.

CBC: complete blood count; TSAT: transferrin saturation; CRP: C-reactive protein; IU: international units; UC: ulcerative colitis; IM: intramuscular; µg: microgram; ω-3 FA: ω-3 fatty acids; UC: ulcerative colitis; and IV: intravenous. Table summary aided by ChatGPT 2025.

## Data Availability

No new data were created or analyzed in this study.

## Supplementary Materials

The following supporting information can be downloaded at: https://www.mdpi.com/article/10.3390/nu18020204/s1, Figure S1: Prisma Flow Diagram [30].
