# Supplementary material for: Approach to Malnutrition and Oral Nutrition Therapy in Adults with IBD: What to Consider
Source: Nutrients. 2026 Jan 8;18(2):204. doi: 10.3390/nu18020204 (PMC12844968; doi:10.3390/nu18020204)
Supplement: Supplementary file 1 [file nutrients-18-00204-s001.zip › nutrients-4015752-supplementary.pdf]

Supplemental Materials

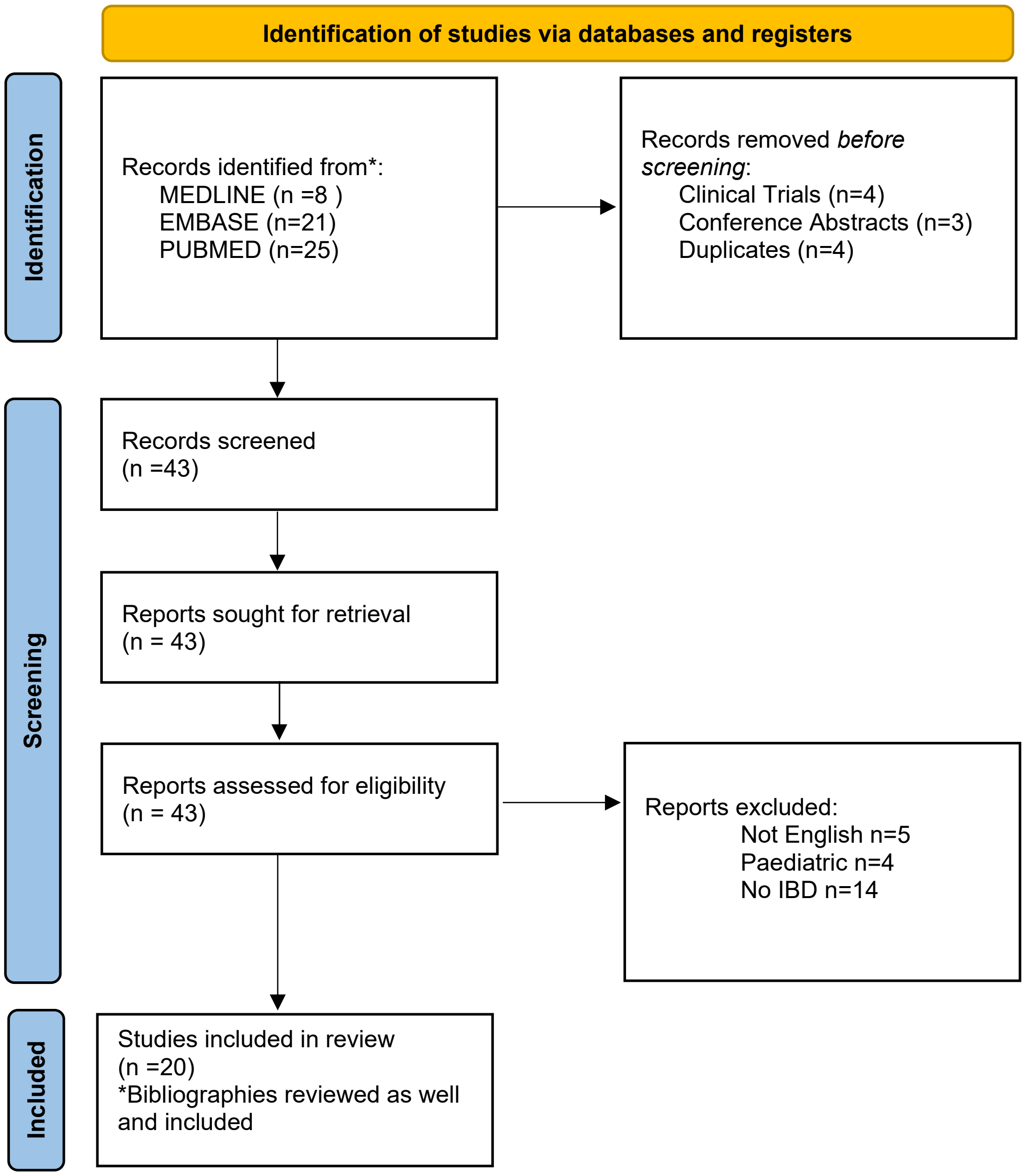

Figure S1. Prisma Flow Diagram.

Source: Page, M.J.; McKenzie, J.E.; Bossuyt, P.M.; Boutron, I.; Hoffmann, T.C.; Mulrow, C.D.; Shamseer, L.; Tetzlaff, J.M.; Akl, E.A.; Brennan, S.E.; et al. The PRISMA 2020 statement: An updated guideline for reporting systematic reviews. *BMJ* **2021**, *372*, n71. https://doi.org/10.1136/bmj.n71.

This work is licensed under CC BY 4.0. To view a copy of this license, visit https://creativecommons.org/licenses/by/4.0/
